# Plasma Metabolomic Profiling Reveals Four Possibly Disrupted Mechanisms in Systemic Sclerosis

**DOI:** 10.3390/biomedicines10030607

**Published:** 2022-03-04

**Authors:** Thomas Bögl, Franz Mlynek, Markus Himmelsbach, Norbert Sepp, Wolfgang Buchberger, Marija Geroldinger-Simić

**Affiliations:** 1 Institute of Analytical and General Chemistry, Johannes Kepler University Linz, 4040 Linz, Austria; thomas.boegl@jku.at (T.B.); franz.mlynek@jku.at (F.M.); markus.himmelsbach@jku.at (M.H.); wolfgang.buchberger@jku.at (W.B.); 2 Department of Dermatology, Ordensklinikum Linz Elisabethinen, 4020 Linz, Austria; norbert.sepp@ordensklinikum.at; 3 Faculty of Medicine, Johannes Kepler University Linz, 4020 Linz, Austria

**Keywords:** systemic sclerosis, metabolomics, LC-MS/MS, ion mobility, kynurenine pathway, urea cycle, lipids, gut dysbiosis

## Abstract

Systemic sclerosis (SSc) is a rare systemic autoimmune disorder marked by high morbidity and increased risk of mortality. Our study aimed to analyze metabolomic profiles of plasma from SSc patients by using targeted and untargeted metabolomics approaches. Furthermore, we aimed to detect biochemical mechanisms relevant to the pathophysiology of SSc. Experiments were performed using high-performance liquid chromatography coupled to mass spectrometry technology. The investigation of plasma samples from SSc patients (*n* = 52) compared to a control group (*n* = 48) allowed us to identify four different dysfunctional metabolic mechanisms, which can be assigned to the kynurenine pathway, the urea cycle, lipid metabolism, and the gut microbiome. These significantly altered metabolic pathways are associated with inflammation, vascular damage, fibrosis, and gut dysbiosis and might be relevant for the pathophysiology of SSc. Further studies are needed to explore the role of these metabolomic networks as possible therapeutic targets of SSc.

## 1. Introduction

Systemic sclerosis (SSc) is a rare autoimmune disease, which is characterized by the production of autoantibodies, vasculopathy, and fibrosis [1,2]. The high morbidity and increased mortality make it a disease of great concern. Patients most commonly suffer skin thickening, digital ulcers, lung fibrosis, pulmonary arterial hypertension, Raynaud’s phenomena, esophageal dysmotility or gut dysbiosis, and produce SSc-related autoantibodies [3,4,5]. The primary cause of death in SSc patients is lung fibrosis followed by pulmonary arterial hypertension or sepsis. The most prominent clinical feature is the fibrosis of the skin and/or internal organs, and skin involvement is a crucial sign for the early diagnosis [4,5]. The modified Rodnan Skin Score (mRSS) was introduced to evaluate skin involvement in SSc patients in clinical trials [6]. Depending on the skin and organ involvement, one can distinguish between limited cutaneous SSc (lcSSc), which manifests in only partial skin (sclerosis of face and distal extremities) and minor systemic involvement, diffuse cutaneous SSc (dcSSc), which includes extensive skin and systemic involvement, and non-cutaneous SSc (ncSSc), with no evident skin involvement [4].

No curative therapy for SSc exists, and in recent years, the vast majority of studies have been published on investigations of new therapeutic strategies [7,8,9,10]. Most of the therapeutic strategies are directed towards inflammatory and vascular pathways, and recently the antifibrotic drug nintedanib has been proven efficient for the therapy of lung fibrosis in SSc [11]. Due to numerous challenges with therapies (efficacy, side effects, multi-morbidity, long-term survival) there is an urgent need to improve existing treatments and detect new possible therapeutic targets for SSc in the future.

Exploring the pathophysiology of SSc is crucial for the improvement of therapy and long-term survival of SSc patients. Metabolites are the final products of the pathophysiologic processes and may play a key role in establishing personalized treatment or biomarkers of drug response in SSc. Metabolomics is a method for describing metabolism by different technologies as published for autoimmune diseases like systemic lupus erythematosus, rheumatoid arthritis, and multiple sclerosis [12]. Mass spectrometry technology holds great potential for a metabolic investigation in a variety of clinical applications as well as different bio-samples [13,14].

Very little is known about the role of metabolites in the pathophysiology of SSc. Over the last five years, several studies were published discovering single metabolites possibly relevant for SSc, and rarely a study would define whole metabolomic pathways involved in the pathophysiology of SSc. In addition, metabolomic studies can sometimes be limited due to the applicability of statistics, which might be problematic due to the rare occurrence of SSc and small study cohorts. Recent data indicated significantly changed metabolites in serum of SSc patients involved in glycolysis, gluconeogenesis, glutamate, and pyruvate metabolism [15]. Analyses of plasma metabolites and fecal microbiota showed glycerophospholipids and benzene derivates to interact with certain fecal bacteria (Desulfovibrio), which may influence gut dysbiosis and inflammation in SSc [16]. Moreover, metabolic profiling of urine from SSc patients revealed deregulated fatty acid oxidation, which might be relevant for inflammation in SSc [17]. A recent study reported dysregulated carnitine in plasma and dendritic cells of SSc patients, and carnitines were suggested to increase inflammation in SSc [18]. Furthermore, altered amino acid metabolism (e.g., betaine, tryptophan, proline, glutamine) was detected in the plasma of SSc patients and has been attributed to changes in vascular endothelial dysfunction and inflammation during SSc [19]. Finally, data have been recently published on characteristic metabolomic changes for certain organ involvement during SSc, like pulmonary arterial hypertension [20] and lung fibrosis [21].

The aim of our study was to analyze metabolomic profiles of plasma from SSc patients by using targeted, and untargeted metabolomics approaches enabled by high-performance liquid chromatography coupled to mass spectrometry technology. Furthermore, we aimed to detect metabolomic networks relevant to the pathophysiology of SSc and generate hypotheses about new therapeutic targets for SSc.

## 2. Materials and Methods

### 2.1. Study Group and Sample Collection

The patients with SSc (*n* = 52) and control group (*n* = 48) were recruited consecutively at the Department of Dermatology, Ordensklinikum Linz in Austria. This study was approved by the Ethics Committee of the Johannes Kepler University Linz in Austria (study protocol number 1265/2019). Diagnosis of the SSc was made according to the criteria of the American College of Rheumatology (ACR) and the European League Against Rheumatism (EULAR) [4]. The inclusion criteria for patients were diagnosis of SSc according to ACR/EULAR criteria and age 18–90. Exclusion criteria for control group were acute infections, liver and/or kidney diseases and diabetes mellitus. Peripheral blood was collected in compliance with the Declaration of Helsinki (1975/83) by using BD-K2-Edta tubes (Fischer Scientific, Schwerte, Germany). After centrifugation of the blood, the resulting plasma was deep-frozen and stored at −80 °C until further treatment.

### 2.2. Sample Preparation

For protein precipitation 50 µL plasma sample is mixed with 150 µL of cooled 5% sulfosalicylic acid followed by 20 min equilibration on a thermo-shaker (4 °C, VWR, Vienna, Austria). Afterwards, samples are centrifuged (8 min, 4 °C, 4200 g, VWR, Vienna, Austria), and 30 µL of the supernatant is transferred to a HPLC vial with a 200 µL glass inlet that also contains 150 µL of acetonitrile as well as 20 µL of an internal standard (Sigma Aldrich, Vienna, Austria, Cell Free Amino Acid Mixture—13C,15N) and is rigorously mixed. The prepared sample is stored at −80 °C until analysis.

### 2.3. HPLC-MS Analysis

High-performance liquid chromatography (HPLC) was performed in a hydrophilic interaction chromatography (HILIC) mode using an XBridge BEH Amide column (2.1 mm × 150 mm, 2.5 μm, Waters, Vienna, Austria) connected to an XBridge Glycan BEH Amide pre-column (130 A. 2.5 μm, 2.1 mm × 5 mm, Waters, Vienna, Austria). HPLC separation was performed by using a gradient of 10 mM ammonium formate and 0.2% formic acid in 18 MΩ-water (solvent A), 0.2% formic acid in acetonitrile (solvent B) and 100 mM ammonium formate with 0.2% formic acid in 18 MΩ-water (solvent C). After injecting 5 µL sample the composition of the gradient was kept constant for 4 min at 4% A and 96% B. Subsequently, solvent A was increased to 18% within 12 min. Then, the gradient was changed within 6 min to a final composition of 40 % A, 40% B and 20% C, which was kept constant for 5 min, followed by switching back to the starting conditions and reconditioning the column for 10 min. These settings were found suitable for measuring polar metabolites such as amino acids and other small molecules.

The targeted method utilized a 1260 Infinity HPLC coupled to a 6460 triple quadrupole mass spectrometer (QQQ-MS) from Agilent Technologies (Waldbronn, Germany). Source parameters for this approach were as follows: gas temperature was set to 325 °C with a flow of 12 L min^−1^, the nebulizer was set to 40 psig, sheath gas was set to 350 °C with a flow of 11 L min^−1^, and the capillary voltage was set to 3500 V.

For untargeted metabolomics a 1290 Infinity HPLC coupled to a 6560 ion mobility quadrupole time-of-flight mass spectrometer (IMS-QTOF-MS) from Agilent Technologies (Waldbronn, Germany) was used. MS/MS experiments for untargeted metabolomics were performed on IMS-QTOF-MS. The IMS was performed by using 4 bit multiplexing with a trap fill time of 3900 µs and a trap release time of 250 µs, and used N_2_ as drift gas. The measurement was done in positive mode in a range of 50 to 1000 m/z. The source settings have been as follows: gas temperature was set to 320 °C with a flow of 10 L min^−1^, nebulizer was set to 45 psig, sheath gas was set to 320 °C with a flow of 10 L min^−1^, and the capillary voltage was set to 4000 V.

### 2.4. Data Pre-Processing

Result files from targeted metabolomics were integrated and processed including an intensity correction with internal standards within the Mass Hunter Quantitative Analysis (Version B.09.00) for QQQ Software (Agilent Technologies, Waldbronn, Germany). Subsequently, results were exported for statistical analysis.

Data obtained by the untargeted approach were initially processed by PNNL PreProcessor (v2019.08.17, Pacific Northwest National Laboratory, Richland, WA, USA). IM Reprocessor (Version 10.00) and IM-MS Browser (Version 10.00) (both Agilent Technologies, Waldbronn, Germany) were used for determining collision cross section ^DT^CCS_N2_ values and applying mass correction. Feature extraction was based on the Mass Profiler Software algorithm (Version B.08.01, Agilent Technologies, Waldbronn, Germany). In the first approach no annotation was performed. After the first statistical analysis, the identification was completed by several steps. First, the exact mass was searched in the Human Metabolome Database (HMDB, accessed on 30 March 2021) [14]. Afterward, matches from HMDB were checked by a comparison of predicted CCS_pred_ values with experimental ^DT^CCS_N2_ values [22]. In this manner, possible matches for each feature were reduced drastically. Subsequently, significant features were identified by matching MS^2^ spectra with entries in the MassBank of North America (MoNA) (http://mona.fiehnlab.ucdavis.edu/, accessed on 30 March 2021) as well as matching calculated CCS_pred_ (http://allccs.zhulab.cn/ [22], accessed on 30 March 2021) values using AllCCS with experimental ^DT^CCS_N2_ values.

### 2.5. Statistical Analysis

Merged results from targeted and untargeted analysis were finally analyzed by the online statistical analysis tool of MetaboAnalyst 4.0. (https/www.metaboanalyst.ca [23], accessed on 5 April 2021). Data were imported into the online platform using missing value estimation, sample normalization by median, data transformation by log transformation, and Pareto scaling. Retrieved *p*-values from univariate analysis were seen as significant if they were below the threshold of 0.05. Additionally, fold changes were seen as significant for up- or downregulation by surpassing an overall increment of 2, respectively. Outcome of these two tests is summarized in a volcano plot. For multi-group comparison, an ANOVA, with associated post hoc analysis, by using Fisher LSD test, was applied. Most noteworthy, dysregulated features are additionally visualized by boxplots. As a further visualization method, a heatmap with an agglomerative hierarchical cluster was chosen. This enables us to present samples or features with similarities close to each other and consequently, to visually recognize clusters. For visualization, Jupyter-Lab 3.2.4 and Python 3.9 were utilized. The following libraries were used: pandas 1.3.4, matplotlib 3.4.3, and seaborn 0.11.2.

## 3. Results

### 3.1. Study Group and Sample Analysis

The cohort of the collected plasma sample included SSc patients (*n* = 52) and the control group (*n* = 48). Females were more dominant in both groups, and the group ratio between females and males was consistent. Most of the SSc patients suffered from lcSSc (*n* = 39) followed by dcSSc (*n* = 11), while the number of ncSSc (*n* =2) was too small for intergroup comparisons. Further clinical information can be retrieved from Supplementary Table S1.

The data from the targeted and untargeted approaches were analyzed separately in the first evaluation. The employed method enabled a large spectrum of interesting pathophysiological molecules to be measured. Metabolites selected by the targeted method mainly consisted of physiologically important small polar molecules, which might need a higher sensitivity for a proper measurement. The targeted and the identified untargeted metabolites were combined for a final statistic. This was performed to generate a list with the most promising molecules for differentiation of the investigated groups.

### 3.2. Targeted Metabolomics

The results of the targeted approach are summarized in Table 1 and Supplementary Table S2. Listed metabolites are significantly up-/or down-regulated in terms of their *p*-values and fold change. Tryptophan was found to be significantly down-regulated, while kynurenine, which is also closely related to tryptophan through the kynurenine pathway (KP), was significantly up-regulated. Alanine, which also can be associated with the KP, was significantly reduced.

In the case of dimethylarginine, which can influence the urea cycle, two forms are known, namely symmetric dimethylarginine (SDMA) and asymmetric dimethylarginine (ADMA). In our approach, both forms could not be separated chromatographically, and therefore, they are presented as the sum parameter “dimethylarginine”, which was significantly up-regulated. Moreover, citrulline and ornithine, both main mediators in the urea cycle, were significantly up-regulated (see Table 1). Additional statistically significant metabolites are given in Supplementary Table S2.

### 3.3. Untargeted Metabolomics

Untargeted metabolomics analyses led to further relevant metabolites from the plasma of SSc patients. All the metabolites from Table 1 from the targeted approach were excluded, and only previously unrecognized features are shown in Table 2. Metabolites found to be regulated were lipids, especially lysophosphatidylcholines (LPCs), sphingomyelins (SMs), metabolites phenylacetylglutamine (PAG), and OH-tryptophan as well as acyl-carnitines (OH-butyrylcarnitine and OH-decanoylcarnitine) (see Table 2). Additionally, observed features with a *p*-value < 0.05 are given in Supplementary Table S3.

### 3.4. Metabolomic Patterns Altered in SSc Patients

**Figure 1:**
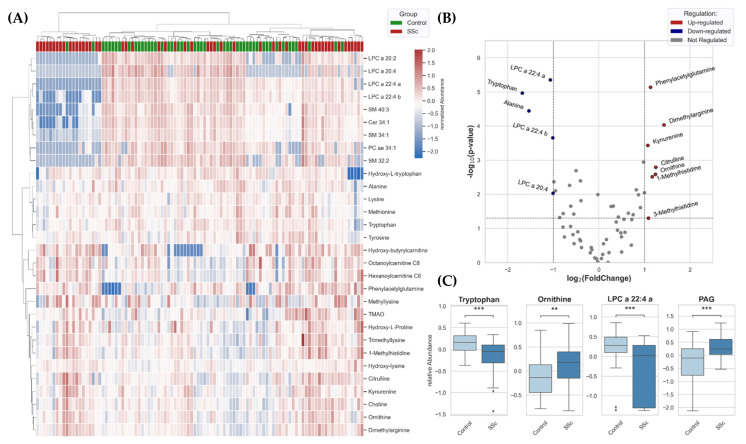
(**A**) Heatmap for all identified features from plasma of SSc patients (*n* = 52) and controls (*n* = 48) was generated based on a selection of the best 30 molecules due to *t*-tests using clustering method (Distance measure: Euclidean, Algorithm: Ward). (**B**) *p*-values and fold change were used for creating a two-dimensional volcano plot of summarized metabolites identified in metabolomics approaches. (**C**) Box Plots (Box = IQR, whiskers = 1.5 × IQR, horizontal bars = median) of significantly dysregulated metabolites. LPC = lysophosphatidylcholine, PAG = phenylacetylglutamine. *p*-values were determined using a two-tailed Student’s *t*-test; *p* < 0.05 was considered significant (** *p* < 0.01, *** *p* < 0.001).

After separate evaluations of targeted and untargeted approaches, the results were combined to gain an insight into the overall data. For a first overview, a heatmap for all identified features was generated based on selecting the 30 best molecules due to *t*-tests, where minor clustering of the SSc samples could be shown (Figure 1A).

*p*-values and fold change were used for creating a two-dimensional volcano plot (Figure 1B). With the applied thresholds, the significantly down-regulated features are depicted on the top-left, and the significantly up-regulated features are depicted on the top-right side. Closely related metabolites can be identified. First, a prominent imbalance of kynurenine and tryptophan was noticed, whereby down-regulation of tryptophan and up-regulation of kynurenine can be observed. Secondly, metabolites of the urea cycle were significantly up-regulated (ornithine, citrulline, and dimethylarginine). Additionally, dysregulation of the lipidome also seems to be present, along with significantly down-regulated LPC species. Another metabolite, phenylacetylglutamine (PAG), was significantly higher in plasma of patients with SSc than controls. Within Figure 1C, the statistically significant differences between metabolites in the plasma of SSc-patients and the control group were emphasized for tryptophan, ornithine, LPCs, and PAG molecules.

### 3.5. Altered Metabolism in lcSSc and dcSSc Patients

**Figure 2:**
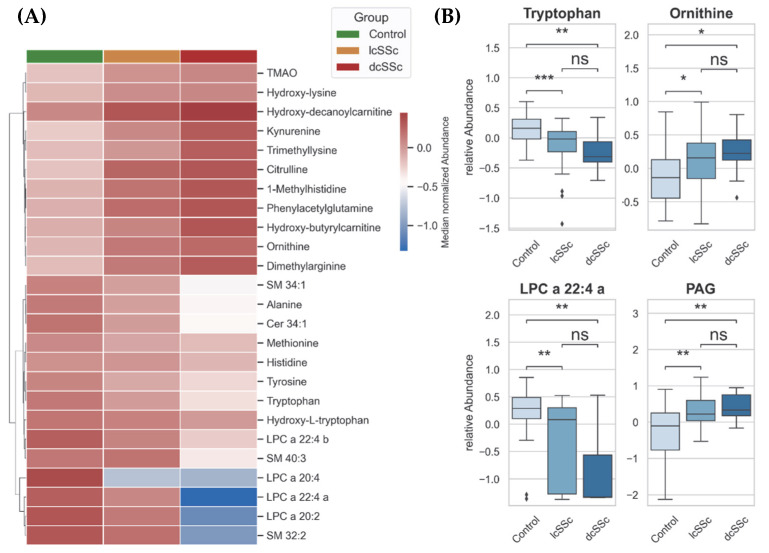
(**A**) Hierarchical heatmap showing a gradient of metabolites from plasma of control group, patients with limited cutaneous SSc and patients with diffuse cutaneous SSc. Distance measure: Euclidean, Clustering-Algorithm: Ward. (**B**) Box Plots (Box = IQR, whiskers = 1.5 × IQR, horizontal bars = median) of significantly changed metabolites: tryptophan, ornithine, LPC = lysophosphatidylcholine, and PAG = phenylacetylglutamine. *p*-values were determined using a two-tailed Student’s *t*-test; *p* < 0.05 was considered significant (* *p* < 0.05, ** *p* < 0.01, *** *p* < 0.001, ns: not significant).

In a further approach, we compared the control group with lcSSc and dcSSc. We excluded ncSSc because a group with only two samples was not suitable for gaining a proper statistical distribution. A comparison of subgroups lcSSc and dcSSc with controls suggested that dysregulations in the observed metabolism were associated with worsening of the disease. This association becomes evident in Figure 2A,B: the metabolites kynurenine, citrulline, ornithine, and PAG are regulated the highest in dcSSc, while the lowest observed concentration is in the control samples.

### 3.6. Cross-Correlation between Lipids and Carnitines

**Figure 3:**
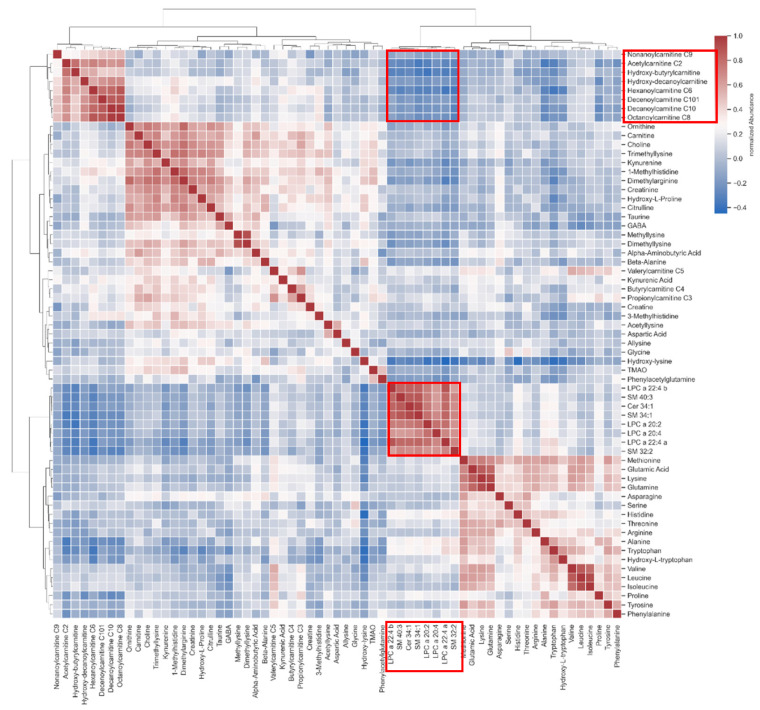
Correlation heatmap showing an interaction of lipids with acyl-carnitines (marked with red squares).

Based on the earlier finding of highly influenced LPC species and carnitine species, a cross-correlation matrix was established to investigate the possibility of statistically insignificant enrichments. Lipid species marked on the x-axis in Figure 3 show a correlation to the acyl-carnitine species marked on the y-axis (marked with red squares within the matrix).

## 4. Discussion

In this study, we explored metabolites from the plasma of SSc patients and a control group by using targeted, and untargeted metabolomics approaches. The main results of our research indicate four possibly disrupted metabolic mechanisms in patients with SSc, namely an enhanced kynurenine pathway, a dysregulated urea cycle, a disrupted lipid metabolism, and a disturbed gut microbiome, which could be involved in the pathophysiology of SSc and might serve as potential targets of treatment for SSc in the future.

**Figure 4:**
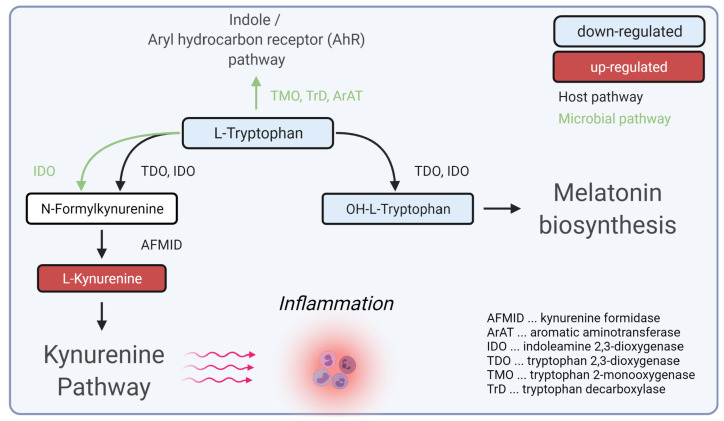
Hypotheses for the role of dysregulated tryptophan/kynurenine pathway during inflammation in SSc. Created with BioRender.com (accessed on 31 December 2021).

We found a statistically significant down-regulation of tryptophan and a statistically significant up-regulation of kynurenine, leading to the conclusion of a dysregulation of the kynurenine pathway in SSc patients compared to controls. Furthermore, OH-tryptophan was also found to be down-regulated within SSc patients. These results implied a depletion of tryptophan towards the kynurenine route, which could result in inflammatory processes (Figure 4).

Previous studies have identified single metabolites of the kynurenine pathway as relevant for SSc [18,19,21,24]. Our data showed these findings combined and identified a dysregulation of the kynurenine pathway in SSc for the first time within one study.

The kynurenine pathway plays an essential role in various diseases such as allergies, autoimmune disorders, or neurodegeneration [25]. Tryptophan catabolism to kynurenine was shown to be regulated by the immune regulatory enzyme indolamine-2,3-dioxygenase (IDO1) [26]. Metabolites of the kynurenine pathway, especially kynurenine, were described to block T-cell proliferation and induce T-cell apoptosis [27,28,29]. It has been shown for several autoimmune diseases that overexpression of kynurenines leads to a dysregulation of regulatory T-cells mediated by pro-inflammatory cytokine cascades [25,30]. Furthermore, local depletion of tryptophan also leads to endothelial cell apoptosis [27,31]. Moreover, tryptophan metabolism is also closely connected to a dysfunctional microbiome in SSc [32,33,34].

As T-cell-mediated inflammation, endothelial cell dysfunction, and gut dysbiosis are essential mechanisms of SSc, the kynurenine pathway might play a crucial role in the pathogenesis of SSc and should further be explored as a possible therapeutic target for the therapy of SSc.

Our data showed up-regulated metabolites ornithine, citrulline in plasma of SSc patients, which are central protagonists in the urea cycle. Ornithine is responsible for the intra-mitochondrial binding of ammonia, whereby it is transformed to citrulline. Citrulline can then pass the mitochondrial membrane through the ornithine translocase (ORNT1) and will again be metabolized to ornithine, which can circulate back into the mitochondria. This metabolization aims to reduce ammonia levels in body fluids by producing urea and arginine. The responsible enzyme is arginase (ARG). ARG catabolizes arginine to ornithine and urea and, in a further stage, to polyamines. Furthermore, NO and citrulline can originate from arginine through NO-synthase (NOS) [35,36].

The overexpression of citrulline was described to indicate the availability of pro-inflammatory stimuli. ARG has been reported to be associated with inflammatory disorders. In psoriasis, an overexpression of ARG leads to decreasing nitric oxide (NO) levels, which modulate the immune responses in tissues [37]. NO dysregulation was identified as having a high impact on the modulation of T-cell responses, with local effects in a tissue due to its short lifetime [35]. Polyamines activity was increased in autoimmune disorders as a pro-inflammatory mediator due to its competition with the cellular methylation for S-adenosylmethionine [38,39].

Furthermore, we found up-regulation of dimethylarginine (DMA) in the plasma of SSc patients. Two different DMAs have been described: symmetric dimethylarginine (SDMA) and asymmetric dimethylarginine (ADMA). In our approach, no separation was achieved. Studies previously showed that ADMA is significantly increased in dcSSc [40,41]. Moreover, it has been shown that ADMA potentially inhibits the degradation of arginine by hindering the catalytic center of NOS [42]. Thus, the up-regulation of DMA might lead to the disruption of the urea cycle (based on an increase in ornithine and citrulline) and subsequently to the downstream production of pro-inflammatory molecules in SSc.

Fibrosis is a common signature of SSc. In this context, generated collagen is dominated by a high content of proline, which is in equilibrium with pyrroline-5-carboxylate (P5C). P5C can be further processed by ornithine aminotransferase (OAT) to ornithine. An association of collagen metabolism with the urea cycle was suggested in metastatic tumor disease [43].

**Figure 5:**
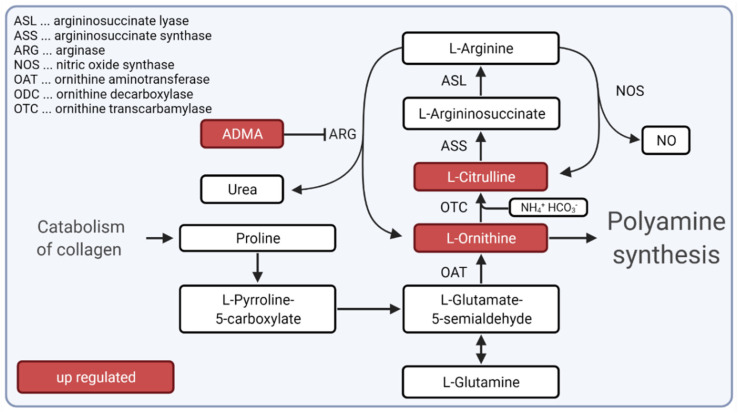
Hypotheses for the role of urea cycle during inflammation and fibrosis in SSc. Our hypothesis suggests that the urea cycle is fueled by collagen catabolism during SSc. Metabolites citrulline and ornithine and dimethylarginine (depicted as ADMA), which is known to inhibit arginase (ARG), were up-regulated in SSc patients. Created with BioRender.com (accessed on 31 December 2021).

We hypothesized that the urea cycle, particularly up-regulated ornithine and citrulline metabolites, could play an important role in inflammation during SSc. Furthermore, we suggest that in SSc, the urea cycle is fuelled in connection with collagen metabolism, whereby citrulline and ornithine concentrations are increased and further stimulate inflammation in SSc patients (Figure 5). Future studies are needed to test the possibility of influencing the urea cycle pathway to block fibrosis and inflammation in SSc.

We detected significant down-regulation of three lysophosphatidylcholines (such as LPC 22:4 a, LPC 22:4 b, and LPC 20:2) in patients with SSc compared to controls. LPCs are part of low-density lipoproteins (LDLs) and are involved in the physiological immune response due to their interaction with Toll-like receptors. It has been shown that LPCs affect oxidative stress, endothelial cells, and lymphocytes and can stimulate pro-inflammatory cytokines. [44] LPCs are chemo-attractants for macrophages and defective clearance through phagocytic cells has been reported to play an important role in systemic lupus erythematosus [45,46]. Our data on LPCs differ from the literature, by showing three down-regulated LPCs molecules indicating that the role of LPCs in SSs might be different for specific LPCs-species. Thus, LPCs might be involved in inflammation and endothelial damage during SSc disease, and further studies are needed to elucidate the role of different LPCs species in SSc and to investigate if LPCs might serve as potential therapeutic targets for SSc in the future.

Our metabolomics study further showed significant down-regulation of sphingomyelins (SM 34:1 and SM 40:3) in the plasma of SSc patients compared to the control group. Previous studies described SMs to be involved in controlling of fibrosis in the skin, lungs, and kidneys [47,48]. Furthermore, our previously published lipidomics study demonstrated significantly decreased SMs in the plasma of SSc patients with more intensive skin sclerosis (dcSSc or mRSS > 14; SM 30:1, SM 32:2, and SM 40:4) [49]. Therefore, it can be suggested that certain species of SMs might affect skin sclerosis of SSc patients. Further studies are needed to determine the possibility of targeting SMs as a new therapeutic strategy for organ fibrosis during SSc.

Our metabolomic analyses showed correlations of lipids such as SMs and LPCs with acyl-carnitines in the plasma of SSc patients. Even though acyl-carnitines are not regulated in a statistically significant manner, the correlation with lipids could indicate their involvement in the pathogenesis of SSc. Acyl-carnitines and lipid metabolism have been reported as dysregulated in autoimmune disorders, especially in combination with disrupted gut microbiome [50].

Acyl-carnitines function as a transporter of fatty acid chains from the cytosol to the inner mitochondria, where fatty acids are further processed by beta-oxidation. Beta-oxidation or fatty acid oxidation (FAO) is a well-known source of energy and, in combination with high glycolysis, FAO is directly linked to collagen production in fibrotic tissue [51,52]. Furthermore, perturbation of FAO has also been reported to be closely related to fibrosis [52,53,54]. Additionally, FAO can also enhance the release of pro-inflammatory cytokines from macrophages. Thus, in addition to LPCs and SMs, the correlation of lipid metabolism with acyl-carnitines, FAO, and the gut microbiome might be relevant for fibrosis and inflammation in patients with SSc and should further be explored in the future.

We identified phenylacetylglutamine (PAG) by the untargeted approach to be significantly up-regulated in patients with SSc compared with the control group. PAG is associated with changes in the gut microbiome, especially in combination with kynurenine pathway metabolites, as presented in our study [55,56]. Kynurenine pathway metabolites can increase due to the synthesis in bacteria, which might enhance cytokine production [30,50,56,57,58]. It has been proposed that fibrosis results in gastrointestinal tract dysmotility, and therefore can have a significant impact on the gut microbiome. Gut dysbiosis is a known feature of SSc [34] and PAG could be an indicator of gastrointestinal involvement [59]. Further studies are needed to explore the role of PAG as a therapeutic target of gastrointestinal symptoms in SSc.

Several clinical trials explored drugs that target metabolites that we found possibly disrupted in our SSc patients, underlining the importance of our data [12,60,61]. Regarding the kynurenine pathway, the quinoline-3-carboxamides like laquinimod and paquinimod, which are structurally similar to kynurenines, have been proven in clinical trials to have positive effects in patients with multiple sclerosis, systemic lupus erythematosus, or SSc [62,63]. Moreover, drugs that influence IDO1 have been shown to have a high potential in treating autoimmune diseases [64]. For example, tocilizumab, which indirectly affects IDO1, has shown positive effects on lung and skin fibrosis in SSc [65]. Regarding the urea cycle, pirfenidone, an ARG1 inhibitor, has shown positive results in patients with idiopathic pulmonary fibrosis, and therefore inhibition of ARG1 might be a valuable therapeutic aim in SSc [66]. On the other side, inhibition of ARG1 can also increase NO, which stimulates inflammation and fibrosis in the lung [67]. Thus, the equilibrium of metabolites is essential for biological effects of metabolic pathways. Further studies, including multi-centre -omics studies, animal models, and in vitro studies, are needed to explore relevant metabolites, their interactions, and their role as possible therapeutic targets in SSc.

## 5. Conclusions

In summary, our data showed four possibly dysregulated metabolic mechanisms in plasma from patients with SSc, namely a dysregulated kynurenine pathway, a dysregulated urea cycle, disrupted lipid metabolism, and a disturbed gut microbiome. An accelerated kynurenine metabolism could induce production of pro-inflammatory cytokines, T-cell-mediated inflammation, endothelial cell dysfunction, and gut dysbiosis in SSc. A dysregulated urea cycle could be involved in inflammation and fibrosis during SSc and might be stimulated due to excessive collagen metabolism, where proline, one of the main constituents of collagen, can increase the production of ornithine and citrulline. A disrupted lipid metabolism, with down-regulated LPCs and SMs in SSc patients, was correlated with acyl-carnitines and FAO, which might stimulate fibrosis and macrophage-mediated inflammation in SSc, especially in combination with a disrupted gut microbiome. Finally, up-regulated PAG could be involved in gut dysbiosis in patients with SSc-related gastrointestinal symptoms.

A limitation of our study is the fact that blood sampling was not done at the same time point during the day and we have no data regarding fasting before blood sampling. Furthermore, due to small numbers of patients in the clinical subgroups, we could not correlate metabolites to single organ involvement or medication in SSc patients. Moreover, our results showed possible changes in metabolic pathways in SSc patients from single cohort study. In the future, multi-centre studies including higher numbers of patients as well as animal-models and in vitro studies are needed to further explore these metabolic pathways and their role as possible therapeutic targets for SSc.

In conclusion, our study of plasma metabolites in patients with SSc identified four possibly disrupted metabolite mechanisms, which are associated with autoimmune inflammation, vascular damage, fibrosis, and gastrointestinal dysbiosis and might be relevant for the pathophysiology of SSc. Further studies are needed to evaluate the role of these metabolomic networks as potential treatment targets for SSc or as personalized biomarkers of drug response in SSc in the future.

## Figures and Tables

**Table 1:** Significantly changed metabolites identified by targeted metabolomics. Mass to charge ratio (m/z), retention time (RT), *p*-value (determined by a two-tailed Student’s *t*-test), and fold change (ratio of SSc to control).

Exact Mass (m/z)	RT/ (min)	Name	*p*-Value	Fold Change (SSc/Control)
205.0971	14.7	Tryptophan	<0.0001	0.3117
90.0549	18.2	Alanine	<0.0001	0.3444
203.1503	23.2	Dimethylarginine	<0.0001	2.6918
209.0921	14.5	Kynurenine	0.0004	2.1049
176.1030	22.1	Citrulline	0.0016	2.3760
133.0972	23.9	Ornithine	0.0026	2.3604

**Table 2:** Dysregulated metabolites were identified by the untargeted metabolomics approach. A feature consists of retention time (RT), measured mass to charge ratio (m/z), and the measured cross collision section (^DT^CCS_N2_). LPC = lysophosphatidylcholine, PAG = phenylacetylglutamine, Cer = ceramide, SM = sphingomyeline, CCS_pred_ was retrieved from AllCCS [22] predictor. *p*-value was determined by a two-tailed Student’s t-test. Fold change was calculated from the ratio of SSc to control group.

Feature (RT_mz_^DT^CCS_N2_)	Metabolite ID	Name	CCS_pred_	m/z_calc_	*p*-Value	Fold Change (SSc/Control)
13.89_572.3679_236.76	HMDB0010401	LPC a 22:4 a (+H)	243.3	572.3711	<0.0001	0.4771
4.283_265.1168_160.02	HMDB06344	PAG (+H)	159.3	265.1183	<0.0001	2.1906
13.511_572.3676_238.65	HMDB0010401	LPC a 22:4 b (+H)	243.3	572.3711	0.0002	0.4958
13.619_673.5254_274.79	HMDB0240612	SM 32:2 (+H)	273.9	673.5279	0.0020	0.7053
15.977_221.0915_150.56	HMDB0000472	OH-tryptophan (+H)	149.7	221.0926	0.0055	0.6392
13.987_548.3692_234.01	HMDB0010392	LPC a 20:2 (+H)	240.7	548.3711	0.0079	0.5156
13.472_685.5613_286.32	HMDB0013464	SM 34:1 (−H2O +H)	283.5	685.5649	0.0101	0.9840
13.203_783.6341_296.53	HMDB0240670	SM 40:3 (+H)	291.2	783.6375	0.0152	0.7605
12.479_248.1485_156.00	HMDB0013127	OH-butyrylcarnitine (+H)	158.4	248.1498	0.0219	1.2434
7.364_332.2419_193.87	HMDB0061636	OH-decanoylcarnitine (+H)	189.3	332.2437	0.0396	1.6815

## Data Availability

The data presented in this study are available on request from the corresponding authors M.G.S. and T.B.

## Supplementary Materials

The following supporting information can be downloaded at: https://www.mdpi.com/article/10.3390/biomedicines10030607/s1, Table S1: Clinical information about sample cohort.; Table S2: Significant regulated metabolites of the targeted approach due to *p*-value (<0.05)., Table S3: Significant regulated metabolites of the untargeted approach due to *p*-value (<0.05).

## Abbreviations

ACR	American College of Rheumatology
ADMA	asymmetric dimethylarginine
AFMID	kynurenine formidase
AhR	Aryl hydrocarbon receptor
ANOVA	Analysis of variance
ArAT	aromatic aminotransferase
ARG	arginase
ASL	argininosuccinate lyase
ASS	argininosuccinate synthase
CCS	collision cross section
Cer	ceramide
dcSSc	diffuse cutaneous Systemic sclerosis
DMA	dimethylarginine
FAO	fatty acid oxidation
HILIC	hydrophilic interaction chromatography
HMDB	Human Metabolome Database
HPLC	high-performance liquid chromatography
IDO	indoleamine 2,3-dioxygenase
IMS-QTOF-MS	ion mobility quadrupole time-of-flight mass spectrometer
KP	kynurenine pathway
lcSSc	limited cutaneous Systemic sclerosis
LDL	low-density lipoproteins
LPC	lysophosphatidylcholine
m/z	mass to charge ratio
MoNA	MassBank of North America
mRSS	modified Rodnan Skin Score
ncSSc	non-cutaneous systemic sclerosis
NOS	nitric oxide synthase
NOS	NO-synthase
OAT	ornithine aminotransferase
ODC	ornithine decarboxylase
ORNT1	ornithine translocase
OTC	ornithine transcarbamylase
P5C	pyrroline-5-carboxylate
PAG	phenylacetylglutamine
QQQ-MS	triple quadrupole mass spectrometer
RT	retention time
SDMA	symmetric dimethylarginine
SM	sphingomyelin
SSc	systemic sclerosis
TDO	tryptophan 2,3-dioxygenase
TMO	tryptophan 2-monooxygenase
TrD	tryptophan decarboxylase
