# Supplementary material for: Plasma Metabolomic Profiling Reveals Four Possibly Disrupted Mechanisms in Systemic Sclerosis
Source: Biomedicines. 2022 Mar 4;10(3):607. doi: 10.3390/biomedicines10030607 (PMC8945346; doi:10.3390/biomedicines10030607)
Supplement: Supplementary file 1 [file biomedicines-10-00607-s001.zip › biomedicines-1561064-supplementary.pdf]

Supplementary Table S1. Clinical information about sample cohort.

**Clinical data**	**Control** (n = 48)	**SSc** (n = 52)	**ncSSc** (n = 2)	**lcSSc** (n = 39)	**dcSSc** (n = 11)
Age[1] / mean ± standard deviation	51 ± 16	60 ± 12	34 ± 9	62 ± 11	57 ± 9
Female / n	37	44	2	36	6
Male / n	11	8	0	3	5
BMI / mean ± standard deviation	-	25 ± 7	26 ± 0	24 ± 7	24 ± 6
**Antibodies**					
Anti-Centromere / n	-	18	1	16	1
Anti-Scl70 / n		17	1	8	8
Anti-Pm-Scl75 / n	-	1	0	1	0
Anti-Rnp/Sm / n	-	3	0	3	0
Anti-Pm-Scl100 / n	-	1	0	1	0
Anti-SSA / n	-	5	0	3	2
**Symptoms**					
Modified Rodnan skin score (mRSS) / mean ± standard deviation	-	12 ± 11	0 ± 0	8 ± 4	28 ± 11
Pulmonary hypertension (PAH) / n	-	10	0	6	4
Lung fibrosis / n	-	14	0	6	8
Dysphagy / n	-	28	1	22	5
Reflux / n	-	27	0	21	6
Raynaud's phenomenon (RP) / n	-	44	2	34	8
Digital ulcers (DU) / n	-	10	0	6	4
Calcinosis cutis / n	-	15	1	11	3
Sicca / n	-	14	0	10	4
**Medication**					
Ambrisentan / n	-	2	0	2	0
Bosentan / n	-	8	0	6	2
Domperidone / n	-	7	0	4	3
Ebetrexat / n	-	14	0	9	5
Ivabradine / n	-	1	0	0	1
Macitentan / n	-	10	0	7	3
Mycophenolate mofetil / n	-	5	0	1	4
Pantoprazole / n	-	26	1	17	8
Prednisolone / n	-	7	0	5	2
Sildenafil / n	-	1	0	1	0
Tadalafil / n	-	1	0	0	1
Treprostinil / n	-	1	0	0	1

[1] p-Values: control/SSc: 0.006; control/lcSSc: 0.002; control/dcSSc: 0.705; control/dcSSc: 0.765; lcSSc/dcSSc: 0.738

Suplementary Table S2. Significant regulated metabolites of the targeted approach according to p-value (<0.05).

Exact Mass m/z	RT min	Name	p-value	FDR corrected p-value	Fold change (SSc/Control)
205.0971	14.7	Tryptophan	<0.0001	0.0005	0.3117
90.0549	18.2	Alanine	<0.0001	0.0009	0.3444
203.1503	23.2	Dimethylarginine	<0.0001	0.0015	2.6918
209.0921	14.5	Kynurenine	0.0004	0.0046	2.1049
176.1030	22.1	Citrulline	0.0016	0.0160	2.3760
133.0972	23.9	Ornithine	0.0026	0.0216	2.3604
170.0924	23.4	1-Methylhistidine	0.0031	0.0218	2.2506
182.0811	16.9	Tyrosine	0.0043	0.0268	0.5033
76.0756	5.2	TMAO	0.0074	0.0408	1.7656
189.1598	23.7	Trimethyllysine	0.0090	0.0451	1.9979
260.1856	4.0	Hexanoylcarnitine	0.0103	0.0467	1.8243
150.0583	15.1	Methionine	0.0222	0.0865	0.6511
204.1230	7.1	Acetylcarnitine	0.0225	0.0865	1.2031
104.1069	5.0	Choline	0.0245	0.0874	1.8805
288.2169	3.4	Octanoylcarnitine	0.0356	0.1103	1.5653
146.1049	23.9	Lysine	0.0364	0.1103	0.5802
246.1699	4.5	Valerylcarnitine	0.0375	0.1104	0.6813
126.0219	14.8	Taurine	0.0458	0.1247	1.3416
116.0706	15.2	Proline	0.0478	0.1247	0.5495
170.0924	23.8	3-Methylhistidine	0.0499	0.1247	2.1241

Suplementary Table S3. Significant regulated metabolites of the untargeted approach according to p-value (<0.05).

Observed Mass m/z	RT min	$^{DT}CCS_{N2}$	p-value	FDR corrected p-value	Fold change (SSc/Control)
287.0993	4.279	166.95	0.000004	0.00048791	2.2385
572.3679	13.890	236.76	0.000004	0.00048791	0.4771
265.1168	4.283	160.02	0.000007	0.00053427	2.1906
577.1323	11.287	222.84	0.000024	0.0013081	3.5230
982.8666	14.994	195.74	0.000061	0.0026669	1.9977
130.0490	4.283	166.63	0.000091	0.0033336	2.0638
627.5318	13.777	265.54	0.000188	0.0058765	0.4985
572.3676	13.511	238.65	0.000221	0.0060392	0.4958
655.1675	20.444	234.20	0.000434	0.0094701	3.1643
880.4826	9.618	298.39	0.000481	0.0094701	0.5783
796.5225	9.653	288.02	0.000508	0.0094701	0.5802
982.8683	14.965	410.95	0.000519	0.0094701	2.8088
188.0699	15.944	150.77	0.000610	0.010283	0.4242
188.0700	15.944	140.20	0.000666	0.010424	0.4130
764.8804	14.778	258.82	0.000805	0.011662	2.0910
502.9284	14.879	218.82	0.000855	0.011662	3.0748
429.1163	11.595	195.05	0.000995	0.011662	1.8714
284.1651	21.624	162.68	0.001031	0.011662	2.7399
844.0756	20.873	245.46	0.001134	0.011662	2.0913
145.0986	17.950	133.49	0.001139	0.011662	1.9719
480.9461	14.653	216.18	0.001210	0.011662	2.6689

77.0915	13.255	170.72	0.001224	0.011662	2.8025
146.0595	15.944	142.36	0.001225	0.011662	0.4548
258.1094	22.914	156.62	0.001387	0.012242	0.5869
982.8685	14.889	303.81	0.001433	0.012242	1.7861
144.0799	15.945	126.83	0.001453	0.012242	0.5421
154.0734	20.872	252.96	0.001646	0.013352	2.3228
429.1163	11.302	195.04	0.001912	0.014954	2.1747
673.5254	13.619	274.79	0.002018	0.015202	0.7053
262.9586	14.910	219.83	0.002082	0.015202	2.7165
666.1790	19.180	236.60	0.002409	0.01702	0.4568
791.1546	6.221	268.60	0.002640	0.017804	2.4636
766.0407	20.443	233.83	0.002683	0.017804	1.5460
229.1817	13.257	156.21	0.002949	0.018912	2.8998
262.9585	14.788	174.00	0.003022	0.018912	2.5756
807.1291	6.213	257.99	0.003604	0.021927	1.6812
520.5067	13.474	249.89	0.004006	0.023708	0.7548
145.0488	10.072	146.57	0.004124	0.023764	1.4501
786.8622	14.771	250.77	0.004715	0.026219	2.0163
478.2900	13.330	213.84	0.004789	0.026219	0.6876
480.9462	14.916	216.21	0.004989	0.026647	2.2446
221.0915	15.977	150.56	0.005455	0.028445	0.6391
146.0595	15.944	130.20	0.006084	0.030985	0.5255
290.2086	23.411	168.24	0.006471	0.032208	2.5050
795.6706	13.101	304.47	0.006776	0.032977	0.7290
244.9477	14.790	174.33	0.007151	0.033462	2.4528
745.6186	13.319	292.15	0.007181	0.033462	0.7238
548.3662	13.987	234.01	0.007904	0.036064	0.5156
526.3264	13.605	227.19	0.009350	0.041788	0.4972
730.1975	20.871	239.93	0.010032	0.043481	1.8360
685.5613	13.472	286.32	0.010126	0.043481	0.9840
783.6341	13.203	296.53	0.015269	0.064307	0.7605
712.9902	21.168	226.25	0.019138	0.078155	1.5972
652.1637	20.442	234.20	0.019271	0.078155	1.4786
786.1998	11.610	257.55	0.020029	0.078295	1.3767
925.9038	24.246	214.25	0.020297	0.078295	1.5893
429.1158	11.585	186.73	0.020439	0.078295	1.7132
240.9509	14.763	183.25	0.021079	0.078295	1.8533
690.1894	4.681	240.60	0.021093	0.078295	1.9778
248.1485	12.479	156.00	0.021929	0.080039	1.2434
548.3655	13.634	234.76	0.022469	0.080666	0.5897
84.0443	21.523	136.14	0.026084	0.092135	1.3957
302.0178	20.545	159.85	0.026683	0.092753	1.7129
638.1842	11.608	235.32	0.028176	0.096415	1.4750
249.0167	23.336	159.53	0.032654	0.11002	0.7342
332.2419	7.364	193.87	0.039612	0.13144	1.6815
319.1668	16.948	174.09	0.041590	0.13594	1.8723
282.1188	17.039	164.31	0.048675	0.15676	1.3247
